# A Machine Learning-Based Tropospheric Prediction Approach for High-Precision Real-Time GNSS Positioning

**DOI:** 10.3390/s24102957

**Published:** 2024-05-07

**Authors:** Jianping Chen, Yang Gao

**Affiliations:** Department of Geomatics Engineering, University of Calgary, Calgary, AB T2N 1N4, Canada; ygao@ucalgary.ca

**Keywords:** LSTM, FFNN, troposphere prediction, deep learning, machine learning, neural network

## Abstract

For high-precision positioning applications, various GNSS errors need to be mitigated, including the tropospheric error, which remains a significant error source as it can reach up to a few meters. Although some commercial GNSS correction data providers, such as the Quasi-Zenith Satellite System (QZSS) Centimeter Level Augmentation Service (CLAS), have developed real-time precise regional troposphere products, the service is available only in limited regional areas. The International GNSS Service (IGS) has provided precise troposphere correction data in TRO format post-mission, but its long latency of 1 to 2 weeks makes it unable to support real-time applications. In this work, a real-time troposphere prediction method based on the IGS post-processing products was developed using machine learning techniques to eliminate the long latency problem. The test results from tropospheric predictions over a year using the proposed method indicate that the new method can achieve a prediction accuracy (RMSE) of 2 cm, making it suitable for real-time applications.

## 1. Introduction

For high-precision positioning applications, various GNSS errors need to be mitigated, including the tropospheric error, which can reach up to a few meters. The troposphere is a layer of the Earth’s atmosphere close to the Earth’s surface. Therefore, the tropospheric delay is a local effect. This layer contains atomic and molecular constituents capable of delaying and bending radio signals. As the bending effect is negligible compared to the delay effect, only the delay effect is considered in this research [1]. The tropospheric delays can be divided, based on physical parameters, into two components: the zenith hydrostatic delays (ZHDs) and the zenith wet delays (ZWDs). The zenith total delays (ZTDs) can be obtained by combining the two components [2]. With accurately known local surface atmospheric pressure, the ZHD can be calculated precisely and subsequently applied as a correction to remove its effect on positioning. Yao et al. proposed a local fusion method from the PPP model as well as a few empirical models with a real-time local troposphere fitting model to improve the accuracy and reliability of local Hong Kong tropospheric prediction [3]. Compared to the ZHD, the ZWD is more difficult to model and determine with high accuracy due to local water vapor variations [4]. In order to separate ZWD from ZTDs, a so-called UNB3m model was proposed by Leandro et al. to model the ZHD as well as ZWD based on a few predefined lookup tables. The model evolved from the original UNB3 model by accounting for possible negative humidity [5]. Better accuracy than the original Saastamoinen model was shown in Leandro et al. [5,6]. In this study, we employ the UNB3m model to characterize the ZHD and subtract it from the ZTD to estimate the wet delays. The International GNSS Service (IGS) has developed zenith troposphere products in the SINEX_TRO exchange format (TRO), with data storage dating back to 1997 [7]. The Center for Orbit Determination in Europe (CODE) has provided products since 2017, with an original sampling interval of 2 h, which was changed later to 1 h starting from 8 January 2023. The latency of the IGS products, however, is 1 to 2 weeks, which makes them unable to support real-time applications.

In the realm of real-time troposphere applications, several regional correction services utilize troposphere components generated using data from densely distributed regional Continuously Operating Reference Stations (CORSs). Examples of such regional services include the QZSS CLAS. However, these services cover only limited local areas due to the availability constraints of dense networks. Other studies have attempted to utilize IGS ultra-rapid products with the Precise Point Positioning (PPP) technique to derive ZTDs for near real-time applications [8,9]. Additionally, efforts have been made using CORSs in China to model tropospheric wet delays and utilize the resulting products to enhance PPP convergence [10]. While real-time estimation of the troposphere has achieved adequate accuracy in water vapor estimation, this approach relies heavily on a solid connection between processing centers and remote stations to provide real-time GNSS observation data, making its reliability contingent on infrastructure dependability. However, observation outages are not uncommon among IGS stations, and the possible long re-convergence caused by outages when employing the PPP technique presents further obstacles to applications [11].

Neural network models are well suited for highly complex non-linear problems and have been utilized across various applications for decades [12]. The widely used types of neural networks include the feedforward neural network (FFNN) and recurrent neural network (RNN). The FFNN possesses a simple structure, with information flowing unidirectionally only (forward) within the network, while the RNN has a bi-directional information flow [12,13,14]. In an effort to overcome the gradient vanishing problem in the traditional RNNs, the long-short term memory (LSTM) method was introduced [15]. GNSS atmospheric correction prediction is a suitable application for adopting the machine learning approach. By applying the machine learning method to conduct real-time troposphere prediction, the latency associated with post-processed troposphere products can be eliminated. Numerous studies have already been carried out to apply neural network approaches to prediction of the effect of the ionosphere, which is another important component in the atmospheric delay affecting GNSS positioning accuracy [14,16,17,18,19,20,21,22,23]. There are research results that consistently demonstrate high prediction accuracy on regional or global VTEC. In terms of applying machine learning to troposphere prediction, some researchers have been using machine learning methods in ZWD prediction. Among those studies, Lu et al. developed a tropospheric delay network (TropNet) to estimate the troposphere over the continental USA [2]. The wet delays used in the research are provided by the Geostationary Operational Environmental Satellite-R series and the global forecast system (GFS). The GFS products are updated every 6 h and continuously predicted forward. An average improvement of 11.9% was demonstrated. However, the prediction process requires the most recent 6 h of data for the next 6 h of prediction, making it complicated for field applications. Li et al. employed LSTM on GNSS-derived ZTDs to enhance the Global Pressure-Temperature (GPT3) model for Antarctica [24]. Zhang et al. investigated wide-area precise tropospheric corrections (WAPTCs) together with those derived from GNSS as well as numerical weather prediction (NWP) across mainland China, and validation shows sufficient accuracy [25]. Li et al. applied a back-propagation neural network (BPNN) with meteorological parameters in mainland China. The training data were based on radiosonde data at 182 sites over 7 years [26]. Bi et al. compared BPNNs and proposed a 1D convolutional neural network (1D-CNN) and concluded that the CNN slightly exceeds that of the BPNN with an RMSE of 2.69 cm in China [27]. Meanwhile, Shi et al. introduced an efficient deep learning approach for estimating troposphere delays for CORSs across China [28]. However, both studies encountered limitations due to availability issues of historical data from the CORSs.

Due to the limitations in the existing approaches, a machine-learning-based approach is proposed in this paper which simplifies the implementation of a machine learning algorithm in tropospheric prediction and can lead to existing post-processed IGS troposphere products to support real-time application. The proposed approach offers a practical and accessible solution to troposphere prediction without the need for continuous long-term data and hard-to-access parameters. Instead, it requires readily available variables including time, local temperature, and relative humidity for the prediction, all of which are widely available from various sources. By utilizing such commonly available information, this approach simplifies the prediction process and enhances its feasibility for applications. This paper has examined two tropospheric delay prediction approaches, one based on the LSTM deep learning method and the other on the FFNN, aiming to enhance the accuracy of long-term local tropospheric delay prediction. By utilizing six years of data spanning from 2017 to 2022 for model training and the entire year of 2023 data for testing, the objective is to leverage extensive historical data to train models and subsequently forecast future ZWD. The proposed model has been applied to predict local tropospheric wet delays over a location in Alberta, Canada. The training data were downloaded from the IGS website, and the weather data were retrieved from the Environment Canada website. Evaluation of the prediction performance includes a comparison with the troposphere products obtained from the Center for Orbit Determination in Europe (CODE) over the last 7 years. This comparison also involves assessing the accuracy of the predictions and the estimation from the PPP method.

The paper is structured as follows: Firstly, the methodology of the FFNN and LSTM machine learning methods, along with associated evaluation techniques, is discussed. Subsequently, test cases and results are presented, followed by an analysis of the performance of troposphere prediction using the proposed approach. Finally, conclusions are drawn, and future work is outlined.

## 2. Methodology

The slant troposphere delays can be retrieved as a byproduct of GNSS positioning. By reversing the mapping function and employing weighted averaging techniques, the station vertical troposphere delays can be generated. GPS-derived precipitable water vapor (PWV) was utilized for predicting precipitation events [29]. PPP technique was employed to derive PWV from zenith wet delay (ZWD). Another case study conducted in Turkey used the Artificial Neural Network (ANN) method to predict troposphere wet delay on selected dates representing summer, winter, and spring [30].

In December 2020, IGS finalized version 2 of the TRO file format in an effort to standardize the exchange format for tropospheric products, which can be traced back to 1997 [7]. The troposphere delay values in the TRO file are usually tropospheric zenith total delays (ZTDs) as well as total delay gradients. The zenith troposphere delays the relation between the ZTD and the hydrostatic component (ZHD) as well as the wet part (ZWD):

(1) ZTD=ZWD+ZHD

The effort to model the ZHD has involved the realization of various models, such as the Saastamoinen model and the UNB3m model [5,6,31].

(2) $$ZHD=\frac{\left[\left(0.0022779\pm 0.0024\right)\right]P_{0}}{f_{x}\left(\phi ,H\right)}$$

(3) $$f_{s}\left(\phi ,H\right)=1-0.00266\cos\left(2\phi \right)-0.00028H$$

Equations (2) and (3) describe the Saastamoinen model. $P_{0}$ is the surface pressure in mbar, and $f_{s}(\phi ,H)$ is the function of geodetic latitude and height. In practice, local pressure at the receiver level is usually unavailable, and a default sea level pressure is applied instead. Therefore, the Saastamoinen ZHD remains constant at a particular location. To account for seasonal variations in zenith troposphere as well as the wet component, the UNB3m model was proposed, incorporating look-up tables for parameter variations across different latitudes. This model was developed by the University of New Brunswick, Canada [5,31].

**Figure 1:**
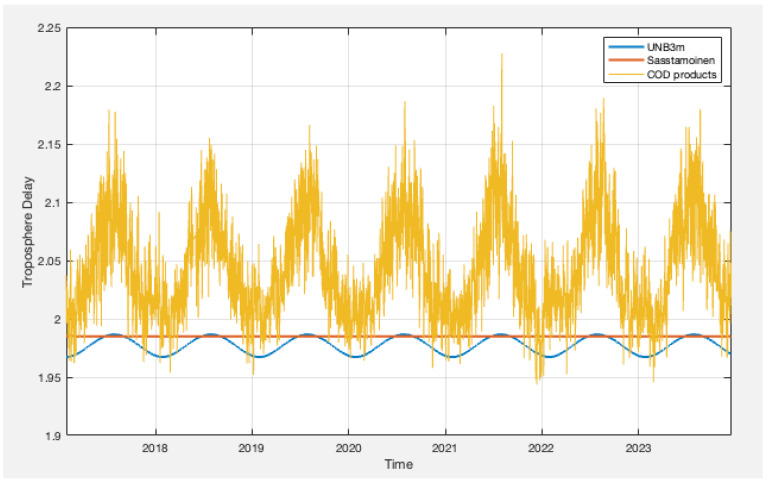
Troposphere comparison between hydrostatic UNB3m, Sasstamoinen, and IGS-COD troposphere products for total zenith troposphere.

In this study, we started with a focus on a single IGS station located near Priddis, Alberta, Canada, with data ranging from 29 January 2017, to 16 December 2023. After analyzing the single station, we subsequently extended the same approach to the additional 11 stations across Canada. Figure 1 depicts the Sasstamoinen model and UNB3m model lines representing only the hydrostatic troposphere component along with the post-processing products over the past 7 years. The Saastamoinen model remains constant throughout the entire period, while the UNB3m model demonstrates a similar pattern to the post-processing products but with a lower magnitude. Notably, the dry part exhibits a larger magnitude during summer months and lower during winters, which is consistent with the observation from the total troposphere from IGS. Hence, the UNB3m hydrostatic part is employed to calculate the wet component of the troposphere.

The converged weights and biases represent the best-fit model for the training inputs and outputs. Subsequently, this model can be utilized to predict unknown outputs based on a new set of inputs. In this study, both a fully connected FFNN and LSTM were employed to predict ZWD. The input list included seasonal and diurnal parameters, which were represented by the quadrature components of the day of the year (DoY) and the hour of the day (HoD). It is commonly known that LSTM is suitable for time sequence prediction, which requires continuous sequences in the training and following validation and prediction periods. However, in our specific application, such continuous data availability poses a challenge, particularly with meteorological data sources of Environment Canada. Therefore, the time inputs are given to both the FFNN and LSTM models. The time components can be described as follows [32]:

(4) $$HoDC=\cos\left(\frac{2\pi \times t}{24}\right),HoDS=\sin\left(\frac{2\pi \times t}{24}\right)$$

(5) $$DoYC=\cos\left(\frac{2\pi \times d}{365.25}\right),DoYS=\sin\left(\frac{2\pi \times d}{365.25}\right)$$

where t represents the hour of the day, and d denotes the day of the year.

The neural networks also make use of temperature and relative humidity as additional inputs. Consequently, the input data dimension is 6 by epochs, while the output data dimension is 1 by epochs.

**Figure 2:**
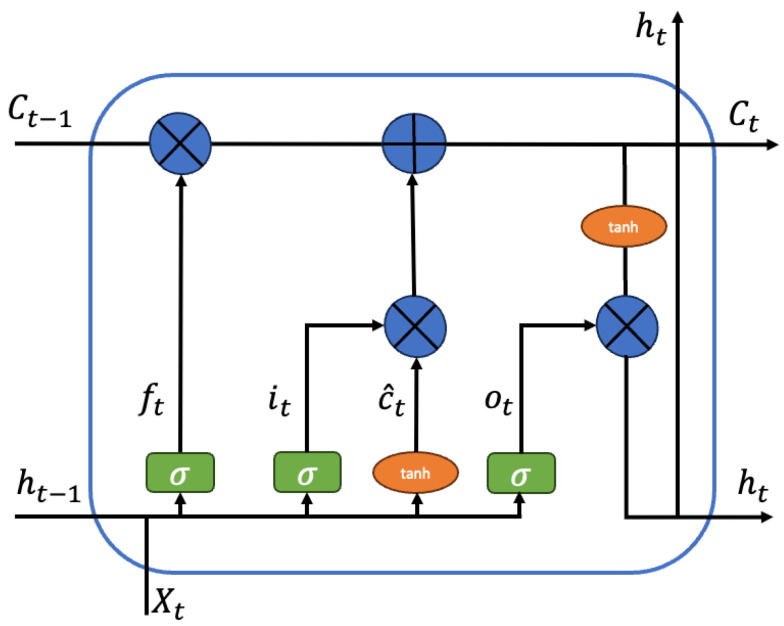
LSTM cell architecture.

The recurrent neural network (RNN) has the advantage of retaining temporal information through its internal loops, which pass the temporal information between different time frames. A chain-like neural network can be depicted by “unrolling” an RNN cell for easy understanding. The LSTM network is a special kind of RNN with characteristics of learning long-term dependency [33]. Figure 2 illustrates the structure of a single LSTM cell. A LSTM cell primarily consists of three gates: the input gate $i_{t}$, the forget gate $f_{t}$, and the output gate $o_{t}$. With the help of the forget gate, an LSTM cell has the ability to retain or forget information to some extent.

Based on the data flow in the architecture, the mathematical model of LSTM can be summarized as [33]:

(6) $$f_{t}=\sigma (W_{f}\left[h_{t-1},x_{t}\right]+b_{f})$$

(7) $$i_{t}=\sigma (W_{i}\left[h_{t-1},x_{t}\right]+b_{i})$$

(8) $${\hat{c}}_{t}=tanh(W_{c}\left[h_{t-1},x_{t}\right]+b_{c})$$

(9) $$c_{t}=f_{t}^{o}c_{t-1}+i_{t}^{o}{\hat{c}}_{f}$$

(10) $$o_{t}=\sigma (W_{o}\left[h_{t-1},x_{t}\right]+b_{o})$$

(11) $$h_{t}=o_{t}^{o}tanh(c_{t})$$

**Figure 3:**
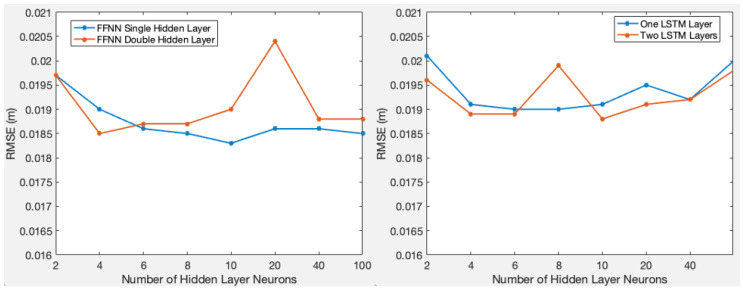
Number of nodes versus validation RMSE for FFNN and LSTM.

Determining the optimal number of hidden neurons in the FFNN hidden layer and the number of LSTM cells in LSTM layers is a trial-and-error process. Too few units may lead to underfitting, while too many units, on the contrary, can cause overfitting [14]. In order to find optimal numbers of neurons or cells for this troposphere research, various sets of numbers were experimented with to determine the most balanced configuration. The whole dataset covers the years from 2017 to 2023, with the year 2023 reserved for testing purposes. Therefore, data from 2017 to 2022 were utilized to achieve this setting verification. Just like regular training and validation, the 80% rule was applied, randomly dividing the dataset into two groups: one training dataset and one validation dataset. A total of 8 settings of 2, 4, 6, 8, 10, 20, 40, and 100 neurons or cells were tested. Figure 3 shows the statistical RMSE of all 8 settings and two options of having a single layer or double layers. Based on the plots, a 10-neuron single hidden layer FFNN and double LSTM layers with 10 cells each were selected for further analysis in this paper. It is important to note that due to the random shuffling of the training dataset, the numbers in the plots may vary slightly each time the test is re-run, but the overall trend remains consistent.

**Figure 4:**
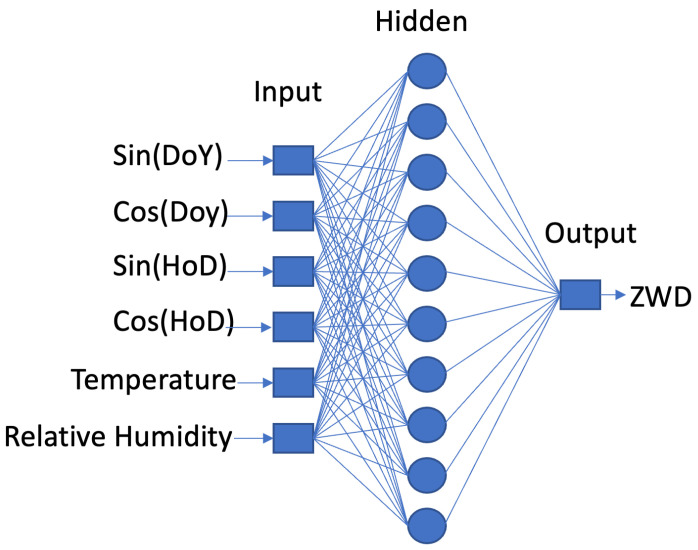
Architecture of FFNN.

Figure 4 shows the architecture of the FFNN model being implemented in this study. As depicted, the model takes 6 inputs: Sin(DoY) (DoYS), Cos(DoY) (DoYC), Sin(HoD) (HoDS), Cos(HoD) (HoDC), temperature, and relative humidity. There is only one item as output: ZWD. One hidden layer resides in the middle with fully connected channels from inputs to output. During the training process, the model utilizes known inputs and outputs to adjust internal weights and biases. Finally, those trained weights and biases enable the model to make predictions. One of the main benefits of using neural networks is their ability to find the relationship between inputs and outputs without explicitly knowing the physical equations.

**Figure 5:**
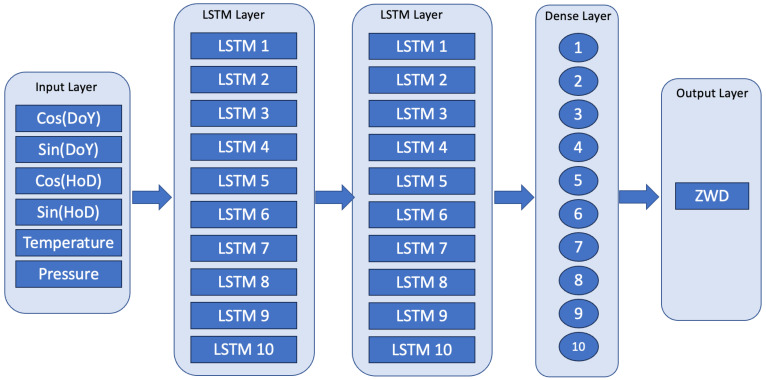
The architecture of LSTM.

Both FFNN and LSTM were chosen for troposphere prediction in this study. The FFNN model comprises one hidden layer with 10 neurons, while the LSTM model consists of two LSTM layers with 10 LSTM cells each. Figure 4 and Figure 5 depict the proposed neural networks for FFNN and LSTM, respectively. 

Figure 4 displays the layers involved in the FFNN model utilized in this study. On the left side are all 6 inputs, while the single output is on the right. Temperature and relative humidity are included to account for local variations, as they affect the actual wet tropospheric delays. All information is transmitted to the output through a 10-neuron hidden layer.

The Levenberg–Marquardt backpropagation algorithm is employed for training parameters. Data division is based on random selection, and MSE is utilized for evaluation purposes.

The LSTM configuration shares the same set of input parameters and output parameters as the FFNN. The input layer transmits information to the first LSTM layer, followed by the second LSTM layer. After the two LSTM layers, a fully connected hidden layer is utilized to connect the dimensions of the output of the LSTM layer with the final output layer. All the LSTM training parameters are listed in Table 1.

## 3. Experiments and Results 

### 3.1. Test Datasets 

To test the troposphere prediction under various conditions, an IGS station (PRDS) located in Priddis, Alberta, Canada, approximately 25 km southwest of the University of Calgary campus, was selected. As CODE started providing troposphere data in 2017, the test data span from the year of 2017 to the end of 2023. The troposphere data from CODE are in TRO format, with a sampling interval of 2 h before 8 January 2023, which was changed to 1 h after that. For compatibility, the resolution of the 2023 data was diluted to 2 h. The TRO files are accessible on the FTP CODE site (ftp.aiub.unibe.ch) (accessed on 16 December 2023). The first day with the PRDS station is 29 January 2017. There are missing data for 10 days in 2018, 3 days in 2019, and 2 days in 2022 for this particular station. Since the troposphere, especially the wet component of the troposphere, has a strong correlation of weather parameters, including temperature and relative humidity, these variables are included in the test datasets. The weather data for Priddis are from Environment Canada website (https://climate.weather.gc.ca) (accessed on 16 December 2023). There is also an outage in the weather data from 1 to 4 September 2020. The entire year of 2023 was used for testing the model, with data available until the data retrieval date of 16 December 2023. The downloaded weather data come with monthly files. The weather data contain temperature and relative humidity information but not pressure. Therefore, only temperature and relative humidity were included in the research. These parameters are also easy to obtain locally if needed for prediction purposes. The time tags in the weather data are in Local Standard Time (LST), which is 7 h behind UTC based on the Canadian Mountain time zone. When combining troposphere and weather data, the weather time tags need to be converted back to UTC. Additionally, the troposphere data in TRO files are total delays, and the wet part needs to be retrieved before feeding into neural networks based on the equation below.

(12) ZWD=ZTD−ZHD

The ZHD is calculated using the UNB3m model. Figure 1 depicts the COD total delay as well as hydrostatic delays from UNB3m and Sasstamoinen. The figure shows that there are instances where negative wet troposphere delays occur. This could be from inaccuracies in modeled hydrostatic delays.

In terms of data splitting for training and validation, we allocated 80% of the data for training and the remaining 20% for validation. Additionally, the data from 2023 were kept aside for testing purposes. It is important to highlight that the training and validation datasets were randomly reshuffled before splitting to prevent any artificially imposed ordering. The time and data details are provided in Table 2.

### 3.2. Accuracy of Tropospheric Prediction

In this study, we evaluated and compared two types of neural networks: the FFNN and LSTM. Due to outages in observing both weather data and troposphere products from CODE and unavailability of local surface pressure data, a dataset of approximately 6-year data was utilized for training and validation, while the data from the entire year of 2023 were for testing. Our analysis included both hourly errors and daily errors. Additionally, the maximum absolute error (MAXAE), the mean absolute error (MAE), and the root-mean-square error (RMSE) were analyzed on both an hourly and daily basis. The absolute error is given by:

(13) $$\left|\epsilon \right|=\left|ZWD_{pred}-ZWD_{tro}\right|$$

where ϵ is the error between the estimate and known zenith wet tropospheric delay values, $ZWD_{pred}$ is the estimated ZWD, and $ZWD_{tro}$ is the reference ZWD from TRO files. The MAXAE, MAE, and RMSE values can be calculated using the following equations:

(14) $$MAXAE=\underset{i=i:n}{\max}\left(\epsilon_{i}\right)$$

(15) $$MAE=\frac{\sum_{i=1}^{n}\left|\epsilon_{i}\right|}{n}$$

(16) $$RMSE=\sqrt{\frac{\sum_{i=1}^{n}{\left(\epsilon_{i}\right)}^{2}}{n}}$$

where the subscript i means each sample point, and n is the total number of samples considered. Total statistics have 2-h intervals, and for daily, this number should be 12 items per day.

**Figure 6:**
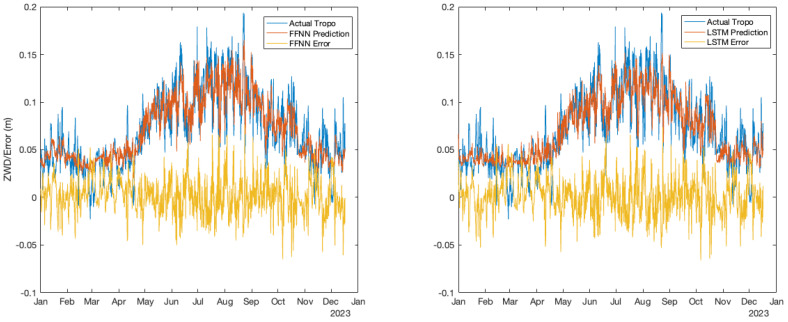
FFNN and LSTM prediction performance. The left side is for the FFNN, and the right side is for LSTM. Actual troposphere products and different neural network predictions as well as the prediction errors are shown in the plots.

For the FFNN, as mentioned in the previous section, a hidden layer with 10 neurons was utilized for both training and prediction purposes. The FFNN training was configured with a predefined target iteration of 1000, and the training was terminated at the 115th iteration. The neural network settings are summarized in Table 3. In Figure 6, a comparison between the neural network prediction and actual troposphere from CODE, spanning the entire year of 2023. It is apparent that the predictions can closely follow the variations but with slightly less magnitude. Overall, the differences between the predicted and actual troposphere are generally small and within a few centimeters.

**Figure 7:**
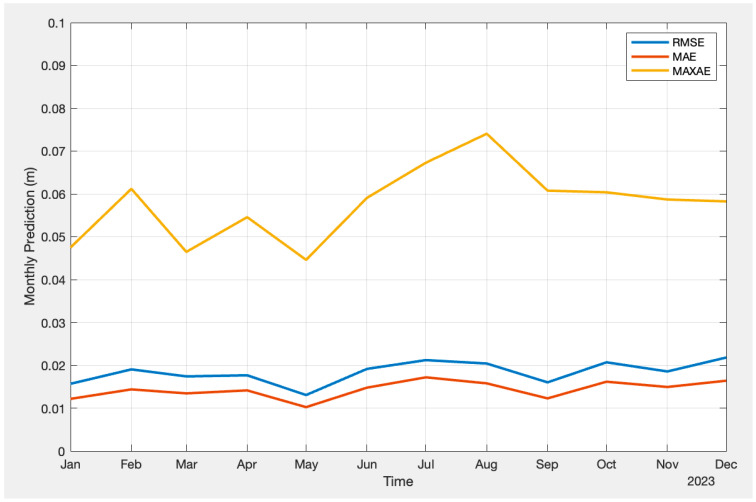
Monthly prediction Results (FFNN).

In Figure 6, the actual difference between the troposphere from CODE and the machine learning prediction is also displayed. In the location of the station, which is very close to the campus of the University of Calgary, it is well known that the temperatures are relatively stable in summer and have more dramatic variations in winter. It is not unusual to have a 30 Celsius temperature change during a one-day timeframe. However, the figure demonstrates that the prediction is not seasonally dependent, as there is no obvious difference between summer and winter. This is relatively unexpected, considering that the temperature changes more significantly in winter than in summer in Southern Alberta. Figure 7 presents the statistical overview of the prediction throughout the entire year from a different perspective. The figure contains RMSE, MAE, and MAXAE for each month of the year of 2023. It is evident that RMSE and MAE remain relatively stable throughout the year, with a magnitude mostly below 2 cm. However, for MAXAE, higher maximum absolute errors are observed during August. 

As for the LSTM model, there are no significant improvements compared to the FFNN model. Figure 6 depicts the comparison between the LSTM prediction and the actual troposphere from CODE.

**Figure 8:**
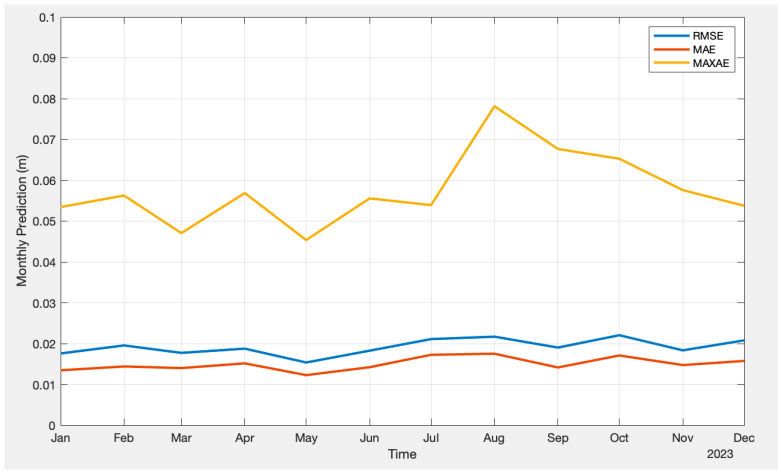
Monthly prediction results (LSTM).

The same figure displays the plot for the difference between the LSTM prediction and the actual troposphere delays from CODE. Again, it demonstrates a relatively small difference compared to the corresponding plot from the FFNN model. Figure 8 provides detailed statistical plots for RMSE, MAE, and MAXAE for individual months of the year 2023.

Table 4 presents the numerical results for the entire year of 2023 from both models. It is observed that the FFNN performs slightly better in terms of RMSE, while LSTM is better with absolute errors.

Based on the single station performance, we extended the analysis to an extended selection across Canada. The stations cover the latitude from 45 degree to 64 degree north. And the longitude covers from coast to coast in Canada. The extra 11 stations are all available from IGS, which has recorded troposphere products since 2017.

**Figure 9:**
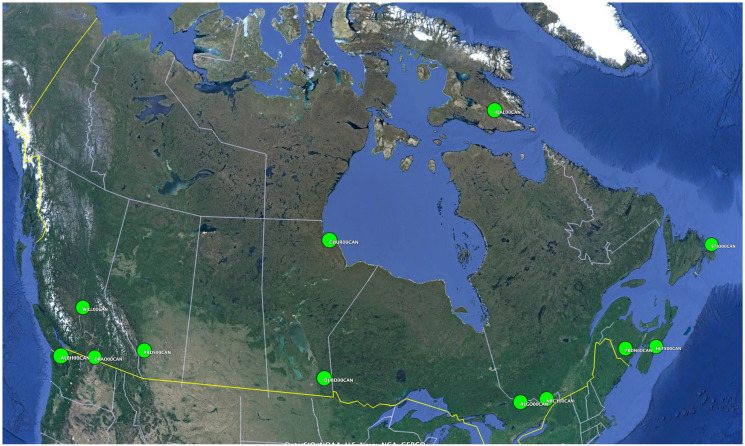
Extra stations included in the analysis.

**Figure 10:**
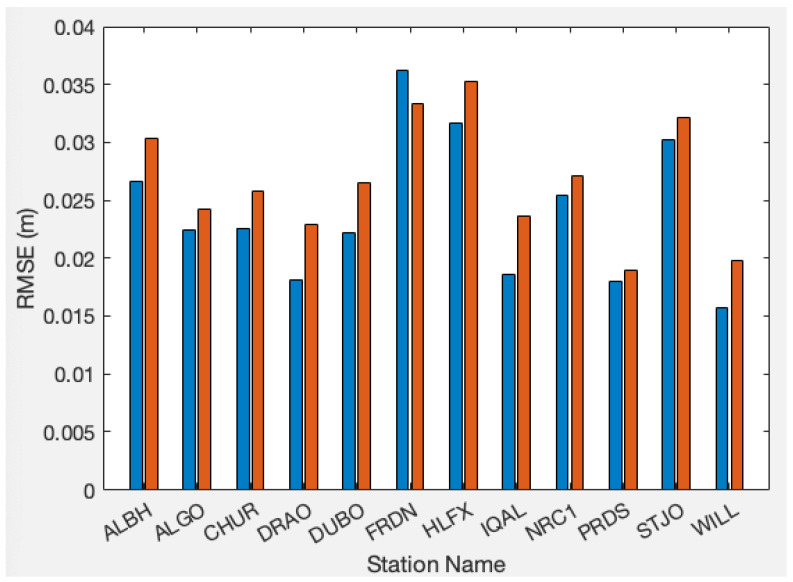
Neural network performance comparison between FFNN and LSTM. RMSEs are showing. Blue bars represent FFNN and red ones indicate LSTM.

The same method, which are 10 nodes for the FFNN and two LSTM layers with 10 neurons each, applies to all 11 extra stations, as shown in Figure 9. The results shown in Figure 10, reveal that the FFNN and LSTM are performing similarly within a few millimeters in RMSE. PRDS from Alberta and WILL from British Columbia demonstrate the lowest RMSE, which indicates the best performance. FRDN from New Brunswick, HLFX of Nova Scotia, and SFJO of Newfoundland and Labrador exhibit poor performances in terms of prediction. Notably, these stations are situated along the Atlantic coast of Canada. One plausible explanation is the sparse distribution of CORSs in the region. 

## 4. Evaluation of Neural Network Models with Precise Point Positioning

In this section, the prediction performance of two neural network models will be assessed by comparing the results to the precise point positioning (PPP) estimation. The observation equations for pseudo-range and carrier phase are shown in the following equations.

(17) $$P_{k}^{j}=\rho^{j}+dt_{r}-dT^{j}+m^{j}T+\left(\frac{f_{1}^{2}}{f_{k}^{2}}\right)I_{1}^{j}+b_{Pk}-B_{Pk}^{j}+\epsilon_{P}$$

(18) $$\Phi_{k}^{j}=\rho^{j}+dt_{r}-dT^{j}+m^{j}T-\left(\frac{f_{1}^{2}}{f_{k}^{2}}\right)I_{1}^{j}+\lambda_{k}N_{k}+b_{\Phi k}-B_{\Phi k}^{j}+\epsilon_{\Phi }$$

where j represents the satellite index, k is frequency, P is the pseudo-range in meters, Φ is the carrier phase in meters, ρ is the geometric range, $dt_{r}$ is the receiver clock in meters, $dT^{j}$ is the satellite j clock, $m^{j}T$ is the slant troposphere delay, $I_{k}^{j}$ is satellite j on frequency k slant ionosphere delay, $b_{Pk}$ is the receiver clock bias for the pseudo-range on frequency k, $B_{Pk}^{j}$ is the satellite j clock bias for the pseudo-range on frequency k, $b_{\Phi k}$ is the receiver clock bias for the carrier phase on frequency k, $B_{\Phi k}^{j}$ is the satellite j clock bias for the carrier phase on frequency k, and ϵ is the measurement error. 

The receiver clock biases for the pseudo-range and carrier phase can be absorbed by the receiver clocks, satellite pseudo-range biases are applied, and satellite carrier-phase biases are ignored. Consequently, the orbit error, clock error, receiver clock error, troposphere, and ionosphere need to be estimated. Since the satellite carrier-phase biases are ignored, the ambiguity integers cannot be restored, and only float solutions are processed.

(19) $$m^{j}T=m_{h}ZHD+m_{w}ZWD$$

where $m^{j}T$ is the total slant troposphere delay, $m_{h}$ is the hydrostatic Niell mapping function, $m_{w}$ is the wet Niell mapping function, ZHD is the hydrostatic zenith troposphere delay, and ZWD is the wet zenith troposphere delay. Rewriting the observation equations, we can obtain the following:

(20) $$P_{k}^{j}=\rho^{j}+dt_{rk}-dT^{j}+m_{h}ZHD+m_{w}ZWD+\left(\frac{f_{1}^{2}}{f_{k}^{2}}\right)I_{1}^{j}+\epsilon_{P}$$

(21) $$\Phi_{k}^{j}=\rho^{j}+dt_{rk}-dT^{j}+m_{h}ZHD+m_{w}ZWD-\left(\frac{f_{1}^{2}}{f_{k}^{2}}\right)I_{1}^{j}+\lambda_{k}N_{k}+\epsilon_{\Phi }$$

The slant troposphere delay can be separated into a hydrostatic part and a wet part based on Equation (13). As the hydrostatic part of the zenith troposphere is known from the UNB3m model, the estimated troposphere from the PPP engine is the zenith wet delay. 

**Figure 11:**
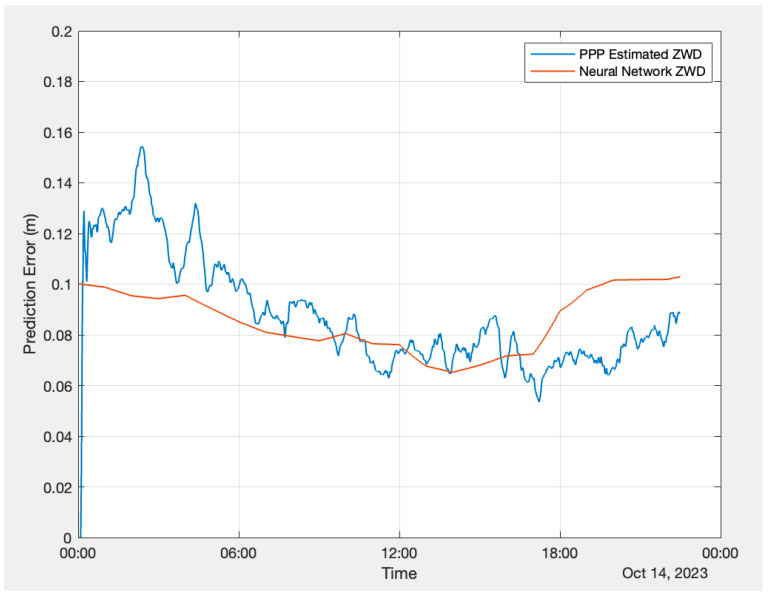
FFNN prediction versus PPP estimation.

**Figure 12:**
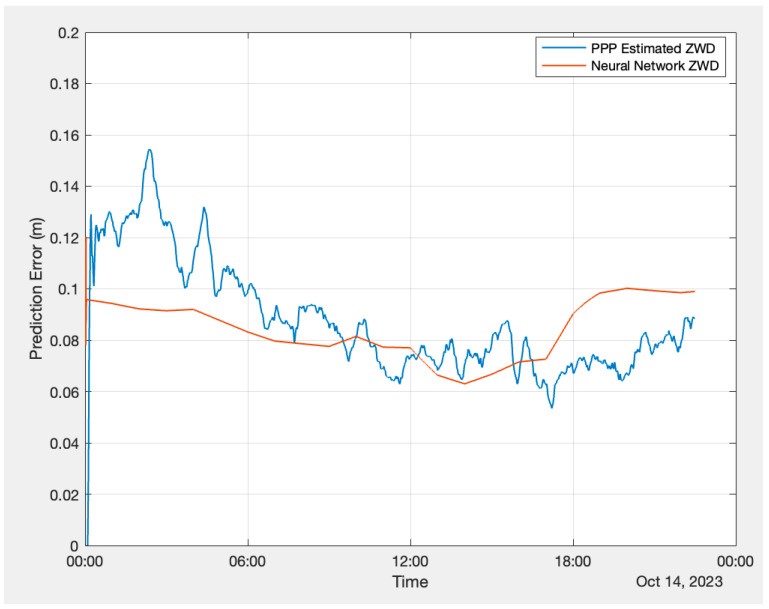
LSTM prediction versus PPP estimation.

To carry out the validation test using PPP, data were logged using a Hemisphere GNSS Phantom 40 board at Hemisphere GNSS’ Calgary office on 14 October 2023. The Hemisphere GNSS P40 board has the capability to track the Hemisphere Atlas LBand signal [34]. The Atlas correction was also logged with the receiver along with observations. Figure 11 and Figure 12 illustrate the performance of the FFNN and LSTM models against the PPP estimation. The plots show that the PPP estimation exhibits more variations compared to the neural network ZWD.

As the PPP Atlas corrections do not include carrier-phase biases, the estimated carrier-phase ambiguities do not converge to integers. Because float PPP requires a longer convergence time, to ensure accurate statistics evaluation, the first 6 h of data were excluded from the analysis, and they are not accounted for in the statistical data in the results. Table 5 below presents the RMSE, MAXAE, and MAE of the FFNN and LSTM performance. Both the FFNN and LSTM models exhibit an RMSE of 1.6 cm and their MAEs are at 1.3 cm level, while the maximum absolute error from LSTM is slightly better than that from the FFNN.

## 5. Conclusions

In this work, we conducted a comparative analysis of a FFNN-based machine learning model and employed an LSTM-based deep learning neural network for long-term troposphere prediction over a duration of one year. Various machine learning settings were tested, and the results indicated that a single hidden layer FFNN model with 10 neurons, as well as a two-LSTM-layer model with 10 hidden cells each, demonstrated the best performance. Both the FFNN and LSTM models achieved RMSE of less than 2 cm for the entire year of 2023. Using real-receiver observation and Hemisphere GNSS Atlas correction, the generated ZWD on 14 October 2023, exhibited an RMSE of approximately 1.6 cm when compared to both FFNN and LSTM predictions. The research suggests that both machine learning methods can serve as simpler alternatives to IGS troposphere products. Unlike other studies that employ machine learning models with more complex inputs and outputs, our proposed models utilize local time and temperature and relative humidity, which are parameters readily available from various sources. Therefore, this method can lead to accuracy of postprocessing troposphere products with slight degradation in real-time applications. Future work will include training with additional aiding information, such as the local air pressure, to further enhance the prediction accuracy. Furthermore, it is essential to examine the predictive accuracy over a dense network and explore potential applications of PPP-RTK.

## Figures and Tables

**Table 1:** Parameters of the LSTM model.

Parameters	Value
Activation Function	Relu
Max Epochs	1000
Number of LSTM Layers	2 or 1
1st LSTM Layer Hidden Units	10
2nd LSTM Layer Hidden Units	10
Number of Dense Layers	1
Learning Rate	0.005
Optimizer	Adam
Dropout	0.2
Loss Function	Mean Squared Error (MSE)

**Table 2:** Time and data details.

	Training Data	Validation Data	Test Data
Time	1 January 2017 to 31 December 2022	1 January 2017 to 31 December 2022	1 January 2023 to 16 December 2023
Size	25,765 × 0.8 = 20,612	25,765 × 0.2 = 5153	4200

**Table 3:** Parameters of the FFNN Model.

Parameters	Value
Data Division	Random
Training Algorithm	Levenberg–Marquardt Backpropagation
Evaluation	Mean Squared Error (MSE)

**Table 4:** Statistics of the two models.

Neural Network	RMSE (m)	MAXAE (m)	MAE (m)
FFNN	0.018	0.075	0.014
LSTM	0.019	0.068	0.015

**Table 5:** Prediction compared to PPP results.

Neural Network	RMSE (m)	MAXAE (m)	MAE (m)
FFNN	0.016	0.037	0.013
LSTM	0.016	0.036	0.013

## Data Availability

As described in the paper, IGS data are from the IGS FTP site, and meteorological data are from the Natural Resources Canada website.
